# Intrusion of the Kuroshio into the South and East China Seas

**DOI:** 10.1038/s41598-017-08206-4

**Published:** 2017-08-11

**Authors:** Chau-Ron Wu, You-Lin Wang, Yong-Fu Lin, Shenn-Yu Chao

**Affiliations:** 1 National Taiwan Normal University, Taipei, Taiwan; 2 University of Maryland, Cambridge, USA

## Abstract

The northward-flowing Kuroshio often intrudes westward and modulates the water masses of the South and East China Seas. These intrusions transcend multiple scales in time and space, which we demonstrate here using various independent data sets. There are two hot spots of intrusion, one in the Luzon Strait and the other off northeast Taiwan, which occur synchronously when the upstream Kuroshio weakens during winter. Beyond seasonal time scales, the two intrusions were not synchronous during 1993–2013. While intrusions into the South China Sea echoed the Pacific Decadal Oscillation, the intrusion northeast of Taiwan decreased markedly before 2002 but regularly reached the shelf thereafter. This change was due to the influence of westward impingements of cyclonic eddies from the open ocean on the Kuroshio main stream in place of anticyclonic eddies. During 1993–2001, decreasing cyclonic eddy impingements moved the Kuroshio away from northeast Taiwan, weakening the Kuroshio intrusion onto the East China Sea shelf. Thereafter, enhanced cyclonic eddy impingement during 2002–2013 weakened the Kuroshio transport, moving it closer to the shelf and enhancing its intrusion into the East China Sea.

## Introduction

**Figure 1:**
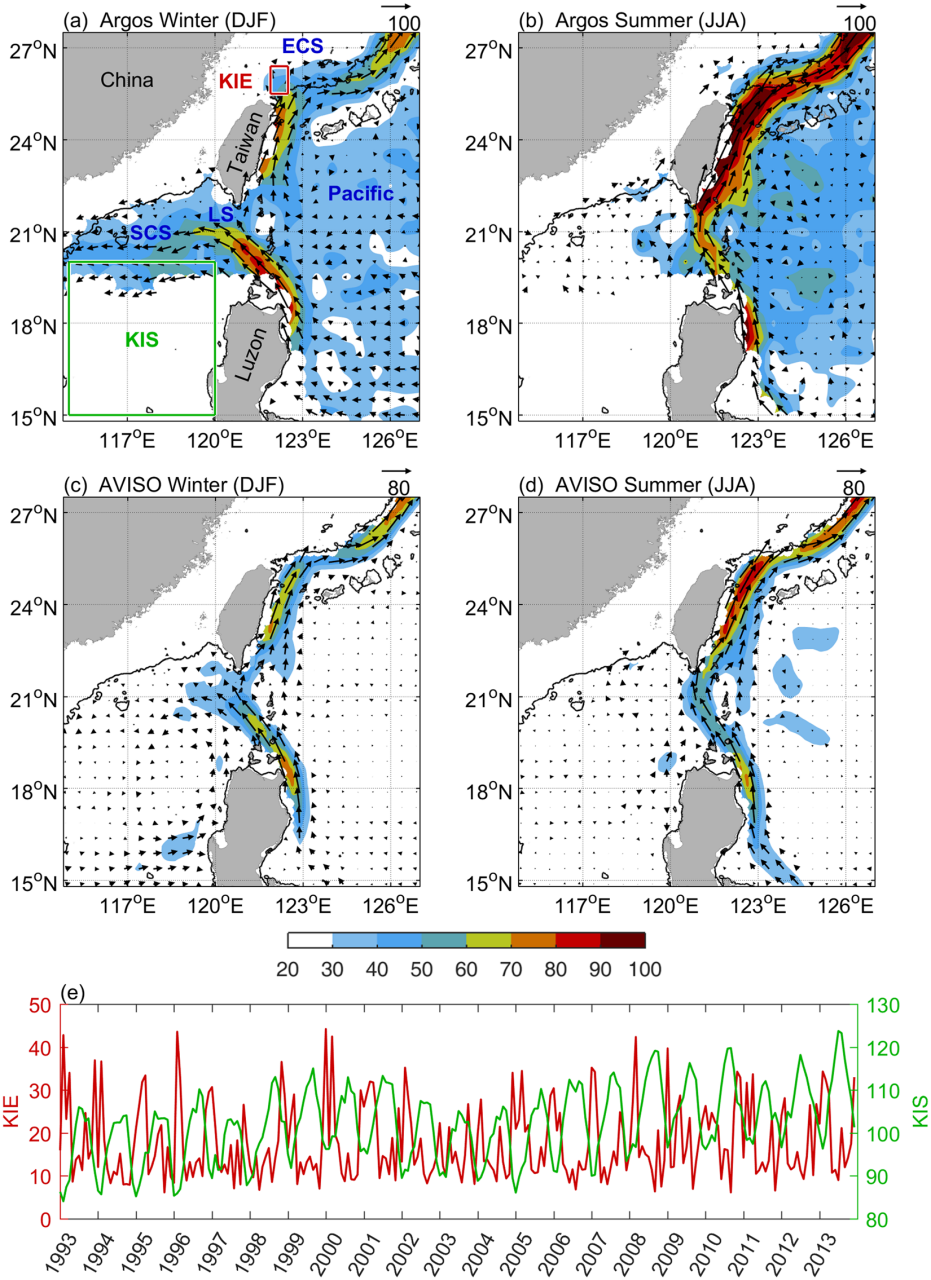
Seasonal Kuroshio intrusions into China Seas averaged over 1993–2013. (**a**) Wintertime (Dec-Jan-Feb) surface currents (vector and shading, in units of cm s^−1^), obtained from Argos measurements (15 m depth). The 200 m isobath (black contour), Luzon Strait (LS), East China Sea (ECS), and South China Sea (SCS) are marked. Boxes KIE and KIS in red and green, respectively are used to index Kuroshio intrusions. Meshes with available gridded data less than 24 months are ignored. (**b**) Same as Fig. 1a, except during summertime (Jun-Jul-Aug). (**c**,**d**) Same as Fig. 1a and b, but for surface geostrophic current derived from AVISO. (**e**) Time series of KIE and KIS index, in units of cm s^−1^ and cm, respectively. All figures are generated with MATLAB (R2017a; https://www.mathworks.com/).

The Kuroshio transports water and heat poleward from the tropics. Along its course, it occasionally intrudes into the South China Sea (SCS) through the Luzon Strait and into the East China Sea (ECS) shelf off northeast Taiwan (Fig. 1a). As a result, water masses are exchanged, influencing mass, heat, salinity, and nutrient balances between the Pacific Ocean and SCS and ECS.

Both the seasonal and interannual variabilities of the Kuroshio intrusions have been documented from observations^1–7^ and models^8, 9^. For example, drifters were found to enter the SCS through the Luzon Strait between October and December, but not between July and September^2^. Recent modeling efforts confirmed that intrusive Kuroshio water is capable of reaching farther westward (~114°E) in winter but is confined to regions east of 118°E in summer. The vertical structure of the intrusive water has also been characterized^8^, and numerical simulations and limited observations have revealed interannual variations. The winter Kuroshio intrusion into the SCS tends to strengthen during El Niño and weaken during La Niña^7^. Using Absolute Dynamic Topography, Wu^5^ demonstrated a strong correlation between Kuroshio intrusion into the SCS and the Pacific Decadal Oscillation (PDO) index. In terms of spatial variations, different types and paths of the Kuroshio intrusion into the SCS have also been identified from satellite altimeter data^3, 6^.

Shipboard Acoustic Doppler Current Profiler (ADCP) observations between 1995 and 1997 indicated that Kuroshio intrusion onto the ECS shelf off northeast Taiwan was dominant in winter, but that the intrusion was relatively inactive in summer as the Kuroshio migrated seaward^4^. Further studies indicated that a strengthened Kuroshio migrating seaward coincided with reduced westward intrusions, and a weakened Kuroshio moving shoreward occurred with enhanced westward intrusions. This documented behavior of the Kuroshio was also confirmed using a high-resolution ocean model and appears to transcend weather-band, seasonal and interannual time scales^9^. Studies to date have only investigated the two intrusion sites separately, and the linkages between them have not been investigated previously.

Two areas, delineated as KIS (115–120°E, 15–20°N)^5^ and KIE (121.9–122.5°E, 25.5–26.4°N)^10^ in Fig. 1a, are used to calculate the index of variability in the Kuroshio intrusion into the SCS and ECS, respectively. The KIS index is the monthly mean sea level (in cm) across the region. A low sea level in KIS (west of Luzon Island) is closely correlated with the Kuroshio intrusion into the SCS^5^. The Kuroshio intrusion current in KIE is generally northeastward with eastward (u) and northward (v) components. The monthly averaged speed index ($\sqrt{{u}^{{\rm{2}}}+{v}^{{\rm{2}}}}$ (in cm s^−1^) in KIE (off northeast Taiwan) accurately reflects the Kuroshio intrusion onto the ECS shelf^10^. Both intrusion indices were used in the investigations described below.

The Kuroshio is driven by Pacific atmospheric circulation. In the past two decades, pronounced changes in atmospheric circulation and surface winds have been observed globally, such as the strengthening of Pacific trade winds^11^. Wind-driven circulation in the tropical Pacific Ocean responded to the intensified trade winds with intensified currents and cooling in the eastern Pacific. Furthermore, tropical ocean circulation affected the Kuroshio via the North Pacific subtropical gyre, and fluctuations in the Kuroshio affected intrusions into the SCS and ECS. This study focused on decadal variations in the intrusions, which remain poorly understood. The intrusions into the SCS and onto the ECS shelf displayed both a clear trend and no distinct trend at different times during the last two decades. Different forcing mechanisms are responsible for the two intrusions. In this study, we demonstrated both seasonal and interannual variations as well as residual trends in both intrusions. On interannual and decadal time scales, the two intrusion sites were mostly independent of each other during 1993–2013. We propose a differential forcing mechanism and discuss decadal trends in the other atmospheric factors causing the intrusion of the Kuroshio onto the ECS shelf.

## Seasonal variability

Figure 1a and b show the Argos velocity composites during winter (Dec–Feb) and summer (Jun–Aug), respectively. Clear seasonal patterns were seen in the area of the Luzon Strait. Under the winter northeast monsoon, the northward-flowing Kuroshio meanders to a significant extent into the SCS (Fig. 1a). In summer (Fig. 1b), the corresponding meander is markedly reduced and confined to a region east of 120°E. Observational data clearly demonstrate this contrasting winter–summer behavior. Intrusive Pacific water can be found west of the Luzon Strait in winter but is less clearly identifiable in summer^2, 7^.

North of the Luzon Strait, the Kuroshio weakens along the east coast of Taiwan in winter (Fig. 1a) but strengthens in summer (Fig. 1b). Based on this behavior, a weakened Kuroshio in winter would lead to its on-shelf encroachment and intrusion, and a strengthened Kuroshio in summer would lead to its seaward migration and decreased intrusion. This anticipated seasonal contrast is too fine in scale to be noticeable off northeast Taiwan in Fig. 1a and b. More specifically, the winter intrusion is widespread over the shelf, but the summer Kuroshio main stream stays off the shelf break (200 m isobath). This seasonal pattern also agrees with the velocity composite of 10-year shipboard ADCP observations^12^.

Figure 1c and d show the altimeter-based geostrophic velocity in winter and summer, respectively. These seasonal flow patterns and intensity were similar to those derived from Argos drifters (Fig. 1a and b), particularly in the area of the Luzon Strait, including the intrusion position and pathway of the Kuroshio. However, the altimeter data indicated a weaker velocity than did the Argos data. The magnitude of the Argos velocity was closer to that measured by both mooring and shipboard ADCPs; the altimeter-based geostrophic velocity was weaker, in agreement with earlier studies in the area^10, 13^.

Figure 1e shows the time series of the two Kuroshio intrusion indices derived from the proxy areas KIS and KIE during 1993–2013. The two series are correlated on a seasonal time scale. However, the indices in KIS and KIE showed monthly to interannual variations, and the overall correlation coefficient (R) decreased to a modest value of −0.41 with a significance level of >99%. The lower-than-anticipated R arose from variations other than seasonal. Focusing on only the seasonal variations, the index in KIS peaked, whereas that in KIE reached a minimum in summer, with the reverse pattern observed in winter. Thus, the two intrusions occurred more or less synchronously on seasonal time scales; however, the mechanisms seem to differ. An intense winter monsoon enhanced the Kuroshio intrusion into the SCS^14^. Northward migration of the North Equatorial Current bifurcation during winter resulted in a weakened Kuroshio off Luzon, also enhancing the intrusion into the SCS^5, 15, 16^. However, the Kuroshio intrusion onto the ECS shelf may be closely correlated with both the zonal surface heat flux gradient and wind stress in the area. The slower Kuroshio off east Taiwan in winter also promoted on-shelf intrusion^10^.

## Interannual variability and decadal trend

**Figure 2:**
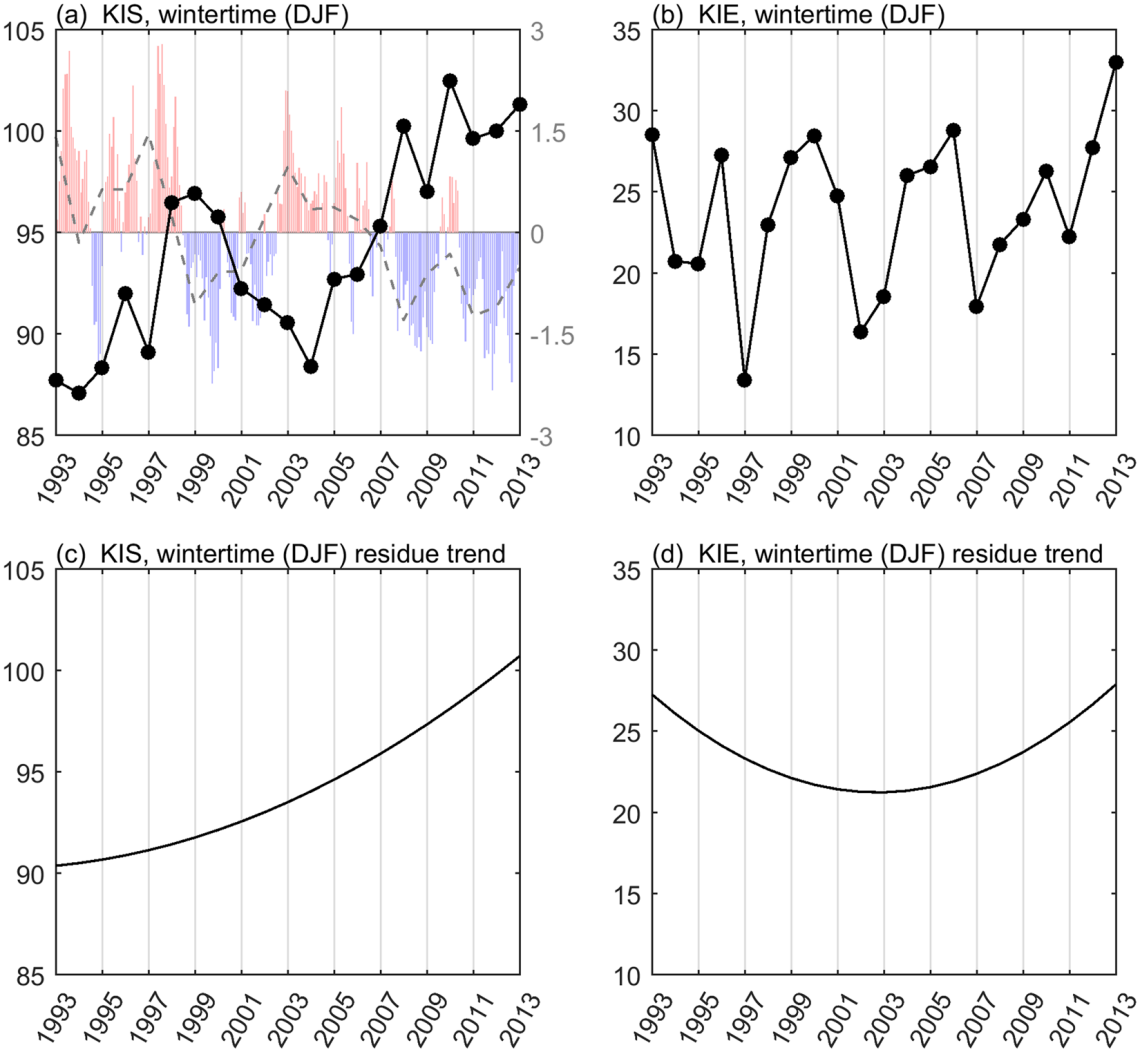
Kuroshio intrusions into China Seas during wintertime (Dec-Jan-Feb, 1993–2013). (**a**) Time series of winter-only KIS index (in units of cm). Positive (negative) PDO indices are also shown in red (blue) colored bars. Dashed line is the yearly mean PDO index. (**b**) Time series of winter-only KIE index (in units of cm s^−1^). (**c**,**d**) Same as the black lines with dots in Fig. 2a and b, except for the residue trend of winter-only KIS and KIE indices, respectively. All figures are generated with MATLAB (R2017a; https://www.mathworks.com/).

Since the intrusion at the two sites often takes place during winter, we focused on the winter-only indices (derived by averaging over the period from December to February of the following year) for KIS and KIE to investigate the interannual and decadal variabilities. Figure 2a and b show the resultant KIS and KIE indices in winter during 1993–2013. At a glance, the winter intrusion into the SCS was indicated by a low KIS index in Fig. 2a, and it intensified during 1993–1997 and 2001–2006 but weakened during 1998–2000 and 2007–2013. These episodes reflect the phase change of the PDO (red and blue bars in Fig. 2a). The annual mean PDO index (dashed line) was correlated with the winter-only KIS index during 1993–2013, with an R value of −0.75 above the 99% significance level. Positive PDO generally coincided with a low winter KIS index and enhanced winter intrusions into the SCS, whereas negative PDO led to the opposite pattern. However, a weak correlation exists between the PDO index and KIE winter index (R = 0.29, with a statistical significance below the 90% confidence level) in Fig. 2b. Thus, the PDO generally failed to indicate the winter Kuroshio intrusion onto the ECS shelf.

In addition, the last intrinsic mode functions (IMFs) for the KIS and KIE indices were extracted using the ensemble empirical mode decomposition (EEMD)^17^ to infer the interannual trends from 1993 to 2013. Also known as the Hilbert-Huang transformation, the EEMD decomposes oscillations with time-varying resolution to preserve nonstationarity and nonlinearity more effectively. It is therefore useful for preserving time-varying time scales intrinsically dictated by the prevailing physics at a specific instant. For each mode of IMF, the number of extrema and zero crossings (after removing the mean) must be equal or differ by no more than one. Each IMF contains linear or nonlinear signals from data and is complete, mostly orthogonal, and adaptive^17, 18^. Starting from either the KIS or KIE index, EEMD successively removed the highest remaining wavenumber IMF from the time series until the last one (the least wavy IMF) emerged. We focused on the tendency of the last IMF shown in Fig. 2c and d. The last IMF of the winter-only KIS index showed a prominent decadal trend with a >11 cm increase from 1993 to 2013 (Fig. 2c). This increase was within the range reported in previous studies^19, 20^. Low sea level in KIS generally favors winter Kuroshio intrusion into the SCS. As the low sea level increases, the Kuroshio intrusion in winter should weaken accordingly.

**Figure 3:**
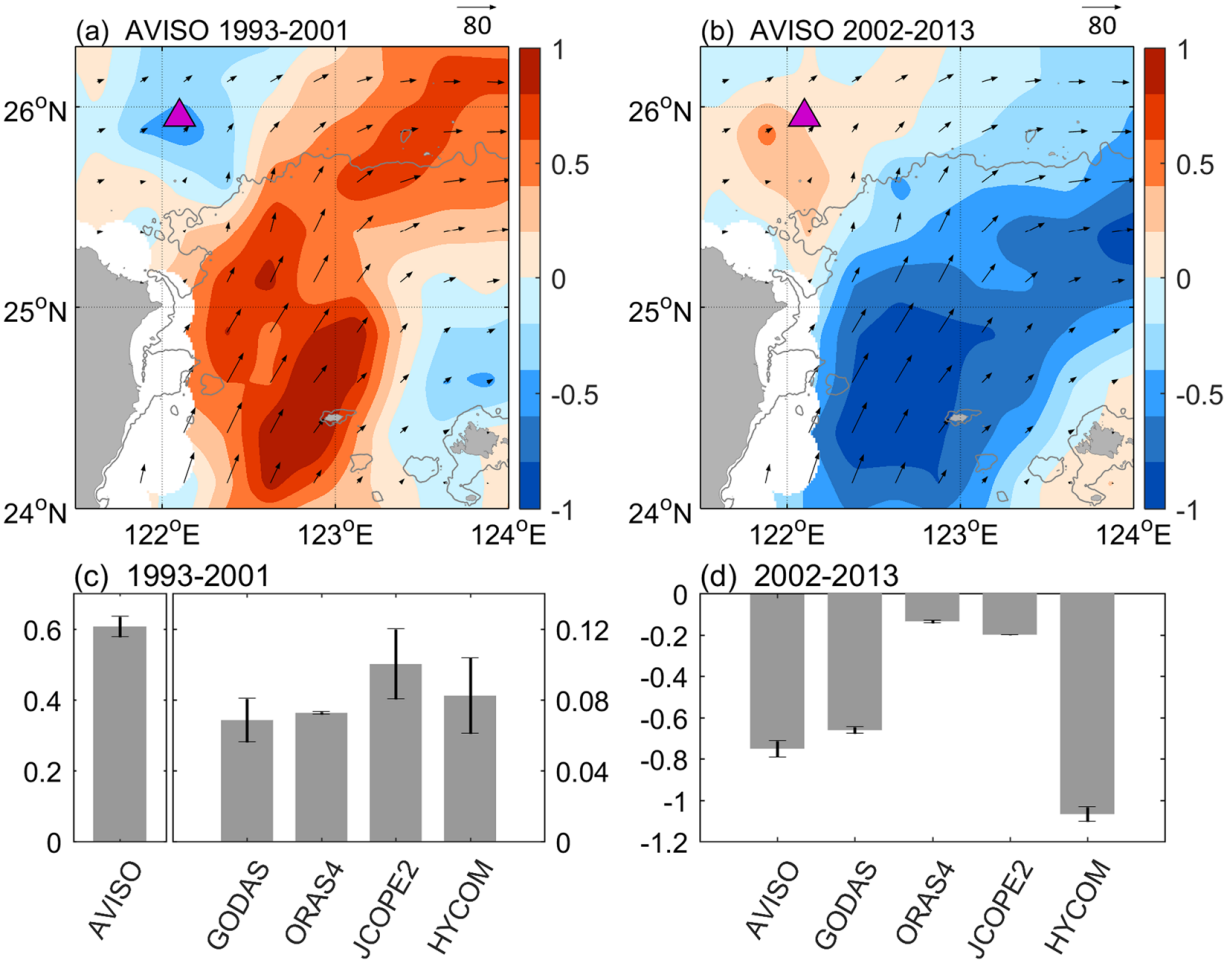
Linear trend of the last IMF of surface velocity off east Taiwan. (**a**,**b**) Linear trend of the last IMF calculated from AVISO altimeter data during 1993–2001 and 2002–2013, respectively. Trend (shading, in units of cm s^−1^ yr^−1^), mean velocity (vector, in units of cm s^−1^), 200 m isobath (gray contour) and the KIE region (triangle) are also shown. (**c**,**d**) The area-averaged linear trend (in units of cm s^−1^ yr^−1^) of the last IMF of the upstream Kuroshio deriving from five independent data sets during 1993–2001 and 2002–2013, respectively. All figures are generated with MATLAB (R2017a; https://www.mathworks.com/).

In contrast to the trend displayed by the winter KIS, the last IMF of the winter KIE initially decreased during 1993–2001 and then increased during 2002–2013 (Fig. 2d). Thus, the winter Kuroshio flowing into the ECS tended to stay away from the shelf before 2002 but became more intrusive thereafter. Based on previous studies, the Kuroshio intrusion in the ECS should be inversely correlated with the strength of the upstream Kuroshio off east Taiwan^10^. To verify whether this inverse relationship transcends interannual time scales, we divided 1993–2013 into two periods, 1993–2001 and 2002–2013. Surface geostrophic currents from satellite altimeter data (AVISO) were used to calculate the linear trend of the upstream Kuroshio intensity for the two periods. The decadal trend was positive during 1993–2001 (Fig. 3a) but negative after 2002 (Fig. 3b), similar to the inverse relationship between incoming Kuroshio strength and on-shelf intrusion on interannual time scales.

Additional data sets also revealed similar tendencies. Surface velocities (near a 15 m depth) were taken from independent data sets that included four ocean reanalysis products (HYCOM, JCOPE-2, GODAS, and ORAS4), each derived from a distinct assimilation method or simulation setting. All data sets consistently showed upstream Kuroshio intensification during the first period (1993–2001) but subsequent weakening (during 2002–2013) with varying magnitudes depending on the data set (Fig. 3c and d). Figure 3c and d correspond to the periods of 1993–2001 and 2002–2013, respectively. For each period, each data set produced a linear fit of the upstream Kuroshio over different areas of coverage (122–123°E and 24–25°N for AVISO, JCOPE2, and HYCOM; 123–124°E and 22–24°N for GODAS; 122–123°E and 23–24°N for ORAS4). Because the data sets differed in resolution, each data set yielded different coverages from the different path width of the mean state of the Kuroshio. However, the area-averaged results from each data set led to the same trends.

## Mechanism

For the residual trend, the two intrusion sites were in phase before 2001, but the southern site became progressively more inactive thereafter, whereas the northern site became more active. The distance between the two intrusion sites is less than 6°. Intervening dynamical process are expected to occur in the area between the two sites, the most likely of which is mesoscale eddy activity. In the area of the Subtropical Countercurrent, the westward-propagating eddies originating from the interior Pacific frequently approach the east coast of Taiwan^21^. Impinging upon the Kuroshio, the mesoscale eddies are capable of modulating the intensity of the Kuroshio. Cyclonic eddies weaken the Kuroshio, whereas anticyclonic eddies strengthen it^22^. Therefore, intervention by mesoscale eddies may change the intensity and frequency of intrusion northeast of Taiwan.

**Figure 4:**
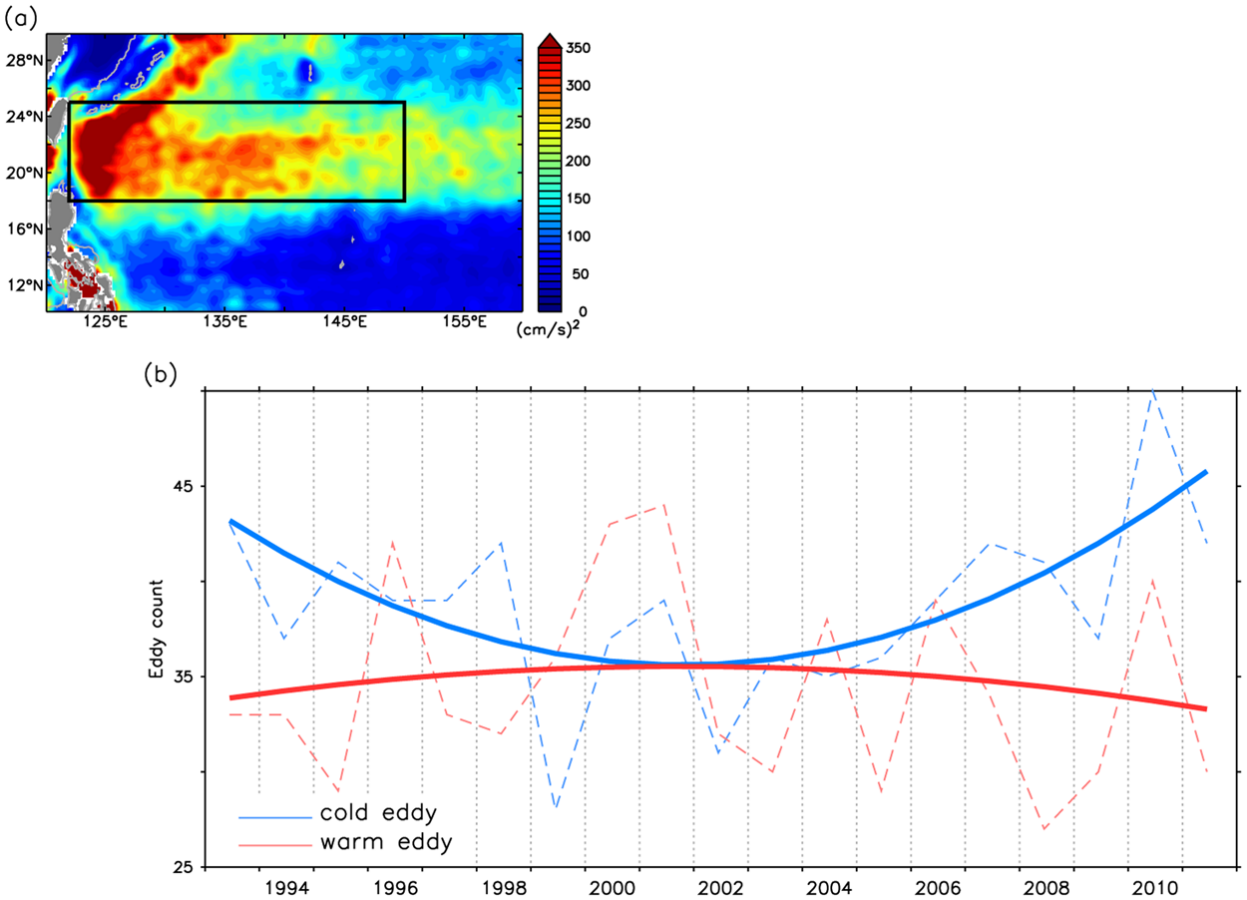
Eddy characteristics in the Subtropical Countercurrent region delineated by a black rectangle (122–150°E and 18–25°N). (**a**) Annual mean of eddy kinetic energy during 1993–2013. (**b**) Yearly cold/warm eddy number. Dash line indicated the number of eddies. Solid line is for the last IMF. All figures are generated with FERRET (v6.93; http://ferret.pmel.noaa.gov/Ferret).

Figure 4 shows the time series of the annual eddy characteristics of each polarity following Chelton *et al*.^23^ in the proxy area of the Subtropical Countercurrent (122–150°E and 18–25°N), indicated by a rectangle in Fig. 4a. The corresponding number of cyclonic (cold) eddies in Fig. 4b decreased during 1993–2001 but increased thereafter, following similar tendencies to the winter KIE index in Fig. 2d. In contrast, the number of anticyclonic (warm) eddies remained generally constant throughout 1993–2011. The increased cyclonic eddy activity from 2002 to 2013 was consistent with the weakening of the Kuroshio and enhancement of its encroachment and intrusion into the ECS.

In addition to the eddy polarity, the residual statistics of eddy strength and the radius of each polarity were also examined. Among these, only the radius of the cyclonic eddies was statistically significant. A monotonic increase in the radius of the cyclonic eddies during 1993–2013 should further weaken the Kuroshio off east Taiwan.

## Atmospheric connection

Several atmospheric factors may also be relevant to the Kuroshio intrusion onto the ECS shelf. Among these, Chao^24^ suggested that local northerly wind-induced shelf-ward Ekman drift in winter enhanced the Kuroshio intrusion. Oey *et al*.^25^ reported that the seasonal change in the Kuroshio appeared to be related to the surface heat flux gradient over the ECS shelf via the “Joint Effect of Baroclinicity and Relief”. A recent study by Wang *et al*.^26^ demonstrated that weakening of the Pacific basin wind stress curl (WSC) reduced the Kuroshio transport and promoted intrusion.

Therefore, we examined the residual trends of local northerly winds, the surface heat flux gradient over the ECS shelf, and basin-wide WSC (data not shown). Among these, the changes in the surface heat flux gradient were not statistically significant during 1993–2013. However, the residual trends of both local northerly winds and basin-wide WSC were fairly constant during 1993–2001 but intensified during 2002–2013. This was consistent with the reinforcement of the effect of the post-2001 increase in cyclonic eddy impingement on the Kuroshio and the associated on-shelf intrusion.

## Conclusion

We analyzed the Kuroshio intrusions into the SCS and onto the ECS shelf using various independent data sets. On seasonal time scales, the intrusions at two sites were generally in phase. However, on interannual and decadal time scales, the two intrusion sites displayed no seasonal correlation during 1993–2013. Different forcing mechanisms were responsible for the two intrusions. The intrusion into the SCS was correlated strongly with the PDO. However, the Kuroshio intrusion onto the ECS shelf was associated with mesoscale eddy activity in the nearby region of the Subtropical Countercurrent. During 1993–2001, decreased cyclonic eddy impingement on the Kuroshio moved the Kuroshio off the shelf break and decreased its intrusion onto the ECS. During 2002–2013, re-strengthened cyclonic eddy impingement led to a weakened Kuroshio, decreasing its on-shelf encroachment and intrusion.

## Methods

### Data set

*a. Current data*. Ocean surface velocities from Argos drifter data provided by Global Drifter Program (GDP, http://www.aoml.noaa.gov) and Archiving, Validation and Interpretation of Satellite Oceanographic data (AVISO version DT-MADT and DT-MSLA, two sat merged of Ssalto/Duacs, http://www.aviso.altimetry.fr) are plotted in Fig. 1. Argos data provide 15 m depth *in-situ* measurements of ocean current. The data have been converted to monthly mean with 0.5° spatial resolution before analysis. Validated AVISO data provide surface geostrophic velocities, ADT, and sea level anomaly that are sampled daily with a spatial resolution of 0.25°.

*b. Ocean mesoscale eddy data*. A statistical dataset of ocean mesoscale eddies is adopted after Chelton *et al*.^23^, which provides parameters such as trajectories, polarity, amplitude and radius.

*c. Wind stress and heat flux data* are from NECPr1 (National Centers for Environmental Prediction/National Center for Atmospheric Research Reanalysis 1)^27^.

*d. The PDO index* ^28^ is downloaded from JISAO (Joint Institute for the Study of the Atmosphere and Ocean, http://research.jisao.washington.edu/pdo/).

### Statistical analyses

The significance test of correlation and trend was performed based on the t-test.

### Eddy kinetic energy

The EKE is based on geostrophic calculation^29^,

1 $$\mathrm{EKE}=\frac{1}{2}({{U^{\prime} }_{g}}^{2}+{{V^{\prime} }_{g}}^{2})\,$$

2 $${U^{\prime} }_{g}=-\frac{g}{f}\frac{{\rm{\Delta }}\eta ^{\prime} }{{\rm{\Delta }}y}\,$$

3 $${V^{\prime} }_{g}=\frac{g}{f}\frac{{\rm{\Delta }}\eta ^{\prime} }{{\rm{\Delta }}x}\,$$

where $U{^{\prime} }_{g}$ and $V{^{\prime} }_{g}$ are the geostrophic velocities, *f* is the Coriolis parameter, and $\eta ^{\prime}$ is sea surface height anomaly from AVISO.
